# Skeleton Precedes Polyp: Visualization of Structural Changes During Coral Growth in *Montipora capricornis*

**DOI:** 10.1002/ece3.72870

**Published:** 2026-01-04

**Authors:** Yixin Li, Chenyi Wang, Zuhong Lu, Chuanliang Wu, Chunpeng He

**Affiliations:** ^1^ Key Laboratory of Environment Controlled Aquaculture (Dalian Ocean University) Ministry of Education Dalian Ocean University Dalian China; ^2^ State Key Laboratory of Coastal and Offshore Engineering Dalian University of Technology Dalian China; ^3^ School of Accounting Dongbei University of Finance and Economics Dalian China; ^4^ State Key Laboratory of Bioelectronics, School of Biological Science and Medical Engineering Southeast University Nanjing China; ^5^ Sanya Coral Reef Ecology Institute Sanya China

**Keywords:** micro‐computed tomography, *Montipora capricornis*, polyp migration, skeletal formation, transit area

## Abstract

Scleractinian corals are foundational to coral reefs, vital marine ecosystems under threat from climate change. *Montipora*, a widely distributed reef‐building genus, contributes through continuous corallum mineralization, yet polyp budding and skeleton formation processes remain elusive. This study elucidates temporal and spatial dynamics of skeletal formation and polyp budding in *Montipora capricornis* using high‐resolution micro‐computed tomography (micro‐CT). We demonstrate that skeleton–canal network formation precedes polyp budding at colony margins, identifying a “transit area” (volumes ~1 mm^3^, skeleton‐to‐void ratio 20%–35%) within tubular canals as a pathway for polyp migration to new calices. This feature serves as a morphological budding marker, enabling visualization of polyp trajectories and growth axes. The polyp‐canal system undergoes dynamic changes, including concurrent skeleton formation and dissolution. These insights establish a structural framework for biomineralization regulation and colony expansion, contributing to the development of coral growth models, and informing environmental impacts on reef‐building in *M. capricornis* .

## Graphical abstract

Using high‐resolution micro‐computed tomography, this study demonstrates that in *Montipora capricornis* , skeleton–canal network formation precedes polyp budding at colony margins, with a “transit area” (volumes ~1 mm^3^, skeleton‐to‐void ratio 20%–35%) in tubular canals serving as a pathway for polyp migration to new calices. This feature acts as a morphological budding marker, enabling visualization of polyp trajectories, growth axes, and dynamic changes involving concurrent skeleton formation and dissolution. These findings provide a structural framework for biomineralization regulation, colony expansion, and modeling environmental impacts on reef‐building corals.

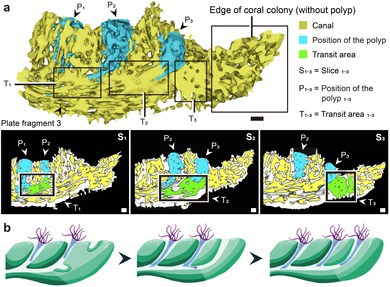

## 1 Introduction

Coral reefs are biodiversity hotspots and natural barriers against coastal erosion (Brandl et al. 2019; Cinner et al. 2016; Woodhead et al. 2019), yet they face escalating threats from climate change, including ocean acidification and warming (Edgar et al. 2023; Smith et al. 2016). Preserving scleractinian corals' growth and reef‐building capacity is essential for ecosystem resilience (Shinzato and Yoshioka 2024).

The skeleton of scleractinian corals is made of aragonite, a polymorph of calcium carbonate, which provides structural support and is essential for the formation of coral reefs (Conci et al. 2024). Thus, biomineralization, as a fundamental process in coral ecology, directly influences coral growth and plays a crucial role in addressing challenges associated with coral reef restoration (Han et al. 2023). It serves as a foundation for understanding how corals respond to environmental changes such as ocean acidification and rising sea temperatures (Levy and Mass 2022). Scleractinian corals reproduce through asexual budding, a process that plays a key role in colony growth by adding new polyps to existing colonies (Guo et al. 2022). This form of reproduction is essential for the expansion of the colony's size, which is vital for reef resilience.

To further understand the processes governing coral growth, recent genomics studies have identified key genes and proteins involved in calcium carbonate deposition (Bhattacharya et al. 2016). These include coral skeletal matrix proteins, coral acid‐rich proteins, and matrix metalloproteinases, all of which contribute to coral skeleton formation (Reggi et al. 2016; Falini et al. 2015; Klepka et al. 2023; Chuang and Mitarai 2020). Coupled with relevant imaging studies, these genomic findings can enhance our understanding of the crucial growth processes involved in coral development (Malik et al. 2021). While much attention has been paid to the molecular and genetic mechanisms of coral growth, there is still limited focus on visually capturing the dynamic processes involved, where biomineralization and budding mechanisms remain poorly understood. Beyond these molecular and genomic perspectives, detailed anatomical descriptions of the soft tissues involved in polyp budding, such as the position and deformation of the coenosarc and associated organs during budding, remain scarce. Most of the available information is inferred indirectly from skeletal structures or low‐resolution surface imaging, rather than from in situ histology or three‐dimensional tissue reconstructions. Previous morphological studies have largely focused on the mineral skeleton and canal network, providing only indirect constraints on the underlying soft‐tissue dynamics. Overall, existing imaging work has mainly highlighted the structural diversity of corals, offering strong evidence for inter‐specific differences, self‐repair mechanisms, and species evolution research (Nir et al. 2011; Kramer et al. 2024, 2022; Fordyce et al. 2020), but much less insight into the real‐time soft‐tissue processes that drive skeletal growth.

To address these challenges, researchers have employed digital image processing techniques to visualize and detect subtle movements of polyps and tissues on the surface of coral colonies (Li, Roger, et al. 2021). This approach provides critical insights into the essential biological and physical functions of coral growth and has implications for understanding the dynamic movement of coral tissue. However, critical growth processes like biomineralization and polyp budding occur within the skeleton–canal network (Li, Liao, et al. 2021). This network consists of the hard skeleton with internal canals filled with coenosarc tissue. These canals connect polyps and facilitate various biological functions. Because of this, it is not possible to obtain detailed visual information through these existing methods and techniques, highlighting their limitations. The skeleton and coenosarc tissues on the surface of the coral colony obstruct in vivo observation of processes like skeleton construction, canal structure changes, and polyp budding or movement, making these studies particularly challenging (Marfenin 2015).

Micro‐computed tomography (micro‐CT) is capable of providing comprehensive structural information in a single acquisition (Urushihara et al. 2016), making it suitable for studying the dynamic growth process relating to delicate skeletal and canal structures hidden within coral colonies (Foster et al. 2016). Micro‐CT is based on X‐ray imaging principles, offering a non‐destructive method to obtain comprehensive structural information of the internal skeleton–canal network in a single acquisition (Li et al. 2020). Compared to traditional methods such as scanning electron microscopy (SEM) and section grinding, which rely on labor‐intensive and structurally destructive sample preparation (Odum and Odum 1955), micro‐CT eliminates the need for such embedding, polishing, or coating steps (Urushihara et al. 2016). In our study, living colonies and fragments were gently blotted to remove excess seawater and scanned in air, allowing direct imaging of the intact skeleton–canal network while keeping the samples viable for subsequent maintenance. This capability facilitates quantitative analysis of coral skeletal microstructure and is becoming increasingly important in the study of reef‐building corals (Crook et al. 2013; Gutiérrez‐Heredia et al. 2015; Mollica et al. 2019; Williams et al. 2024; Corbera et al. 2021).

According to the information provided in The World Register of Marine Species (WoRMS), *Montipora* is one of the largest and most diverse genera among scleractinian corals, with approximately 85 known species, making it the second most species‐rich coral genus after *Acropora*, widely distributed throughout Indo‐Pacific reef ecosystems (Geng et al. 2017). Its widespread presence and role as a reef builder make it an ideal model for studying coral growth patterns and biomineralization processes. Understanding the colony growth patterns, polyp budding mechanisms, and biomineralization processes of *Montipora* is crucial for insights into coral reef formation and resilience. In this work, we select *Montipora capricornis* (Veron 2000) from this coral genus as the research subject. In our previous study on this species, we found that *M. capricornis* belongs to the complex coral clade and is distinguished by an elaborate canal system comprising three types of canals (Li et al. 2023). These include calices for polyp accommodation, mesh‐like canals connecting adjacent calices, and tubular canals beneath the calices interconnecting all calices within a colony. The mesh‐like and tubular canals constitute an integrated network that facilitates essential biological processes within a coral colony. Its structural complexity and the close connections among polyps make it easier for us to obtain coral growth information through the reconstruction of the skeleton and canals.

Using high resolution micro‐CT on nine *M. capricornis* samples (four colonies, five fragments; Table 1), we reconstructed three‐dimensional (3D) skeleton–canal networks and polyp‐canal systems at active margins. We also performed visual modeling of dynamic processes such as polyp budding and calices construction to elucidate the involved growth patterns. These models allow us to analyze and summarize the sequence of skeleton formation and polyp budding. We also investigated structural changes in the skeleton–canal network during growth, providing a theoretical basis for research on coral biomineralization. We identified morphological budding markers in the canals of *M. capricornis* . In this study, we use the term “morphological budding marker” to refer to a recurrent skeletal structure that indicates where a polyp has budded or has been translocated within the colony. In some genera, such as *Pocillopora* and *Stylophora*, polyp movement trajectories can be inferred directly from colony‐scale skeletal and canal architectures (Li, Liao, et al. 2021). For example, in the colony of *Pocillopora damicornis* , polyp budding has occurred at every inverted conical inter‐septal space, and therefore these inverted conical inter‐septal spaces are considered morphological budding markers for *P. damicornis* (Li et al. 2020). Based on these markers, we can trace polyp movement and budding patterns, enabling the visualization of growth axes related to coral directional growth. This discovery deepens our understanding of polyp budding and migration dynamics within *M. capricornis* colonies, providing a foundation for biomineralization research and environmental response modeling.

**TABLE 1:** Parameters of the micro‐CT tests.

Samples	Sample size	Voltage	Current	Resolution	Duration	Number of images	Image width	Image height
*M. capricornis* (colony 1)	25 × 20 × 15 cm	190 kV	250 μA	49 μm	1000 ms	1400	3990 pixels	4000 pixels
*M. capricornis* (colony 2)	20 × 20 × 15 cm	190 kV	250 μA	48 μm	1000 ms	1400	3990 pixels	4000 pixels
*M. capricornis* (colony 3)	25 × 25 × 20 cm	220 kV	300 μA	110 μm	500 ms	1600	2024 pixels	2024 pixels
*M. capricornis* (colony 4)	23 × 22 × 17 cm	220 kV	210 μA	47 μm	1000 ms	1500	3990 pixels	4000 pixels
*M. capricornis* (plate fragment 1, from colony 1)	3 × 3 × 0.7 cm	120 kV	90 μA	14 μm	1000 ms	1600	3990 pixels	4000 pixels
*M. capricornis* (plate fragment 2, from colony 2)	3 × 3 × 0.7 cm	170 kV	100 μA	15 μm	500 ms	1600	2024 pixels	2024 pixels
*M. capricornis* (plate fragment 3, from colony 3)	3 × 3 × 0.7 cm	200 kV	100 μA	18 μm	500 ms	1600	2024 pixels	2024 pixels
*M. capricornis* (plate fragment 4, from colony 4)	3 × 3 × 0.7 cm	120 kV	90 μA	9 μm	500 ms	1700	3300 pixels	2500 pixels
*M. capricornis* (plate fragment 5, from colony 4)	3 × 3 × 0.7 cm	120 kV	90 μA	9 μm	1000 ms	1400	3990 pixels	4000 pixels

## 2 Result

### 2.1 Growth Zones and Dynamic Skeleton Formation in *M. capricornis*

**FIGURE 1:**
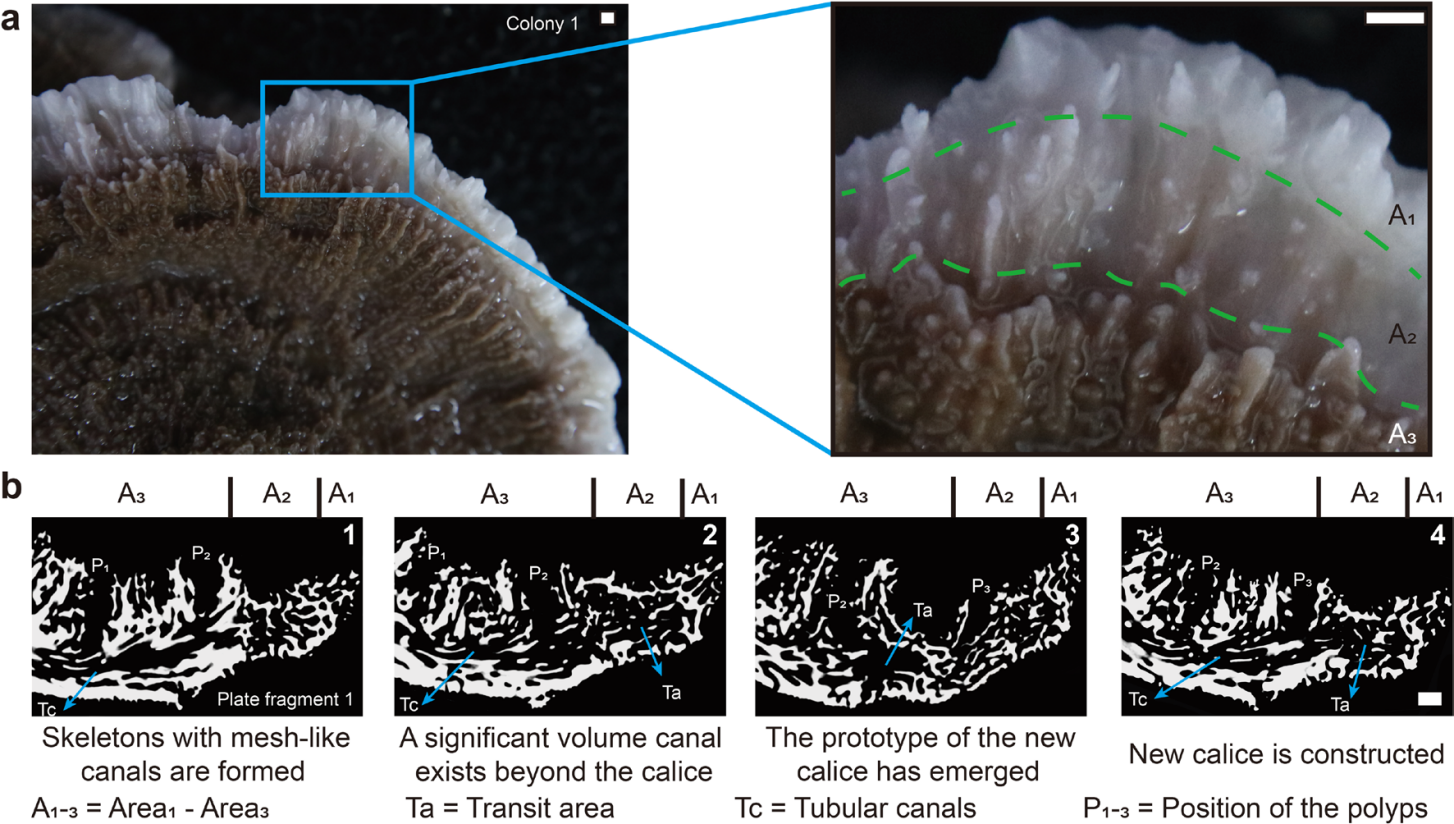
Morphological characteristics and 3D reconstructions of the *M. capricornis* edge zone. (a) The outermost growth area of *M. capricornis* can be categorized into three distinct color zones with varying hues, and the variation in color is primarily attributed to the presence or absence of coral polyps on the semi‐transparent skeletal surface and within the canal system. (b) Transit areas (Ta) and adjacent tubular canals (Tc) are indicated by separate arrows. Calices containing polyps are numbered (P_1_‐P_3_) to clarify their spatial relationships with the underlying canal system. Based on the reconstruction of the skeleton–canal network, our findings indicate that the structural changes during coral growth can be delineated into four distinct stages: Formation of an outermost layer composed solely of skeleton with mesh‐like canals, emergence of large transit areas, development of new calices, and establishment of transit areas between the newly formed calices and adjacent ones. Scale bars: 1 mm.

The growth area at the margin of *M. capricornis* can be distinctly divided into three zones (Figure 1a). The variation in color of the colony edge is mainly due to the presence or absence of coral polyps within the calices or canal system. The outermost zone is the white translucent skeleton (Zone A_1_), with a width of approximately 1 mm. The area near the center of the colony (Zone A_3_) exhibits the color of symbiotic zooxanthellae due to the presence of polyps and coenosarc tissue with zooxanthellae within the canal system or on the surface. Between A_1_ and A_3_, there is a color gradient zone of about 3 mm (Zone A_2_), where the canal system within the translucent skeleton contains newly budded polyps in the process of being transported, with a color similar to that of Zone A_3_ but is comparatively lighter.

**FIGURE 2:**
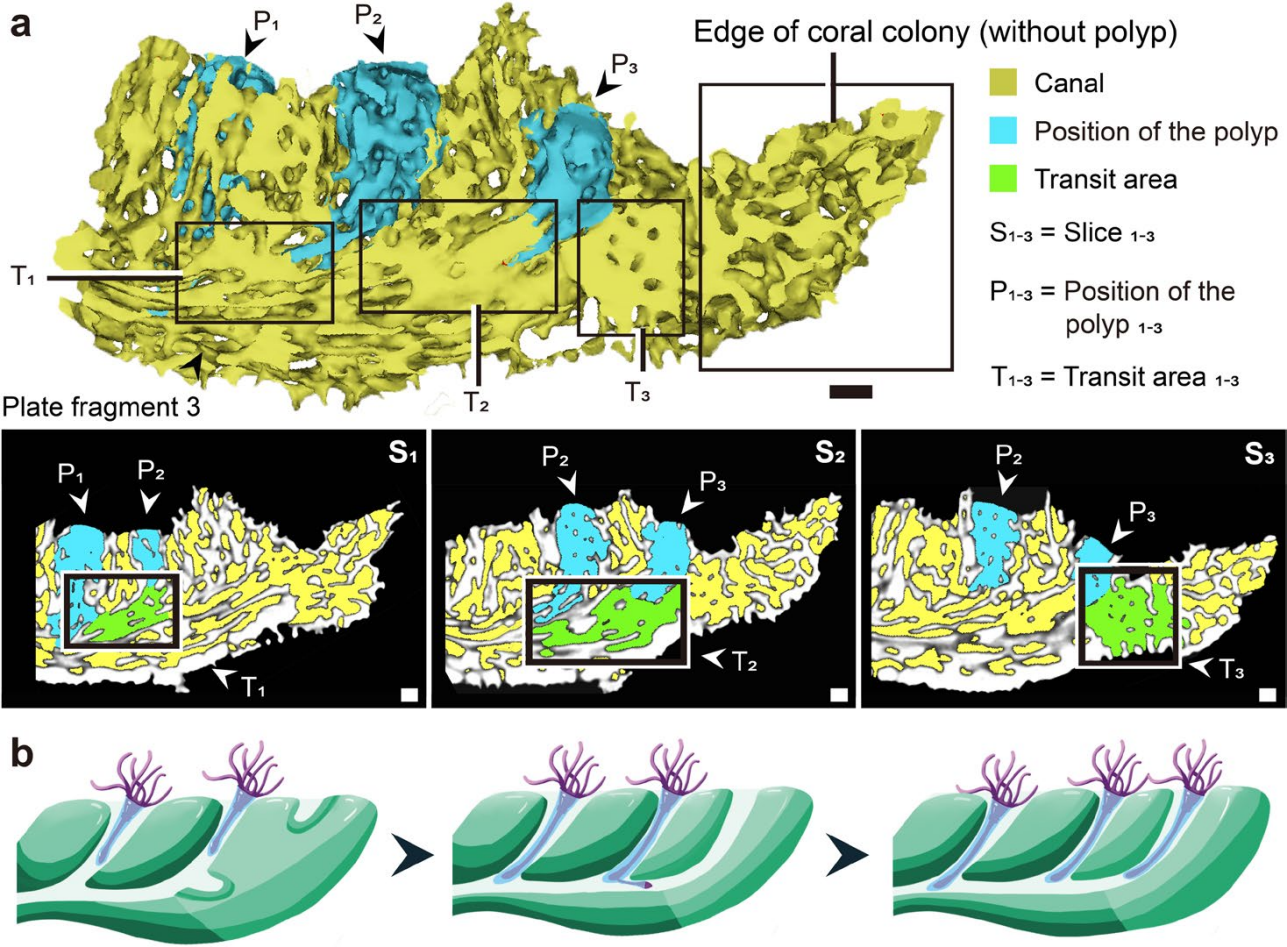
Reconstructions of skeleton–canal network reveal the dynamic growth process of *M. capricornis* . (a) The polyp‐canal system shown in this figure is reconstructed from plate fragment 3 (Table 1), representing an active growth margin. Three calices (where polyps were actually observed in vivo) are distributed in the skeleton–canal network, and the edge of the colony contains only skeletons and mesh‐like canals. S_1_, S_2_: The transit areas distributed between the bottom of (S_1_) calices P_1_ to P_2_ and (S_2_) P_2_ to P_3_. S_3_: The transit area exists in the front of calice P_3_, close to the colony edge. Three representative transit areas (T_1_‐T_3_) are highlighted between successive calices along the growth axis. Each transit area was defined as a contiguous canal enlargement that (i) connects a pair of calices with an inferred budding relationship, (ii) has a void volume at least three times larger than the median volume of adjacent tubular canal segments, and (iii) exhibits a skeleton‐to‐void ratio within the 20%–35% range characteristic of transit areas. (b) Schematic drawing showing the skeleton formation and polyp budding processes at the edge of the coral colony. Scale bars: 1 mm.

We reconstructed the skeleton structures and polyp‐canal systems at the edge of *M. capricornis* colonies using high‐resolution micro‐CT. The 3D reconstructions revealed the presence of mesh‐like canals and tubular canals, both integral to coral growth. The mesh‐like canals connect adjacent calices, while tubular canals form an interconnected network beneath the calices. We observed larger voids within some tubular canals (volume ≈1 mm^3^, skeleton‐to‐void ratio 20%–35%) located between adjacent calices, which we refer to as transit areas (Ta in Figure 1b, T_1‐3_ in Figure 2). These structures may serve as pathways for the movement of newly budded polyps, inferred from their size and position in the canal network. In samples where new calices are present, the transit areas appear to have integrated into the tubular canal, suggesting that after polyp establishment, the structure may have been modified, though this is inferred from the observed skeletal patterns. This allowed us to visualize their growth, reveal skeleton formation patterns, and simulate the dynamic growth process, including polyp budding and movement (Figure 1b).

In Figure 1b, the outermost calices (P_1_–P_3_) are connected by tubular canals (Tc), within which enlarged transit areas (Ta) are present on the side facing the colony margin. The growth process is divided into four stages. In the first stage, calices are absent in Zones A_1_ and A_2_, with only mesh‐like canals within the skeleton. In the second stage, a larger space emerges within the skeleton in Zone A_2_, connecting to the outermost calice in Zone A_3_. In the third stage, initial calice formation begins in Zone A_2_, and the large space in the canal system from the second stage extends toward the new calice. In the fourth stage, the new calice is completed at the edge of Zone A_3_ and connected to the calice from the second stage through the large canal. Our observations suggest that the skeleton‐canal network is dynamically constructed, with evidence of repeated cycles of polyp budding and establishment, inferred from the pattern of canal structures and calice distribution. During this process, Zones A_1_ and A_2_ continue expanding, initiating a new round of growth that perpetuates this cycle within coral colonies.

### 2.2 3D Visualization of Polyp‐Canal Systems

We reconstructed 3D polyp‐canal systems to visualize patterns during coral growth processes such as colony branching, skeleton formation, canal extension, and polyp budding. The reconstruction of the polyp‐canal system in a *M. capricornis* fragment revealed the regulation of polyp budding and skeleton formation patterns (Figure 2). Three sequentially arranged calices, designated as P_1_ to P_3_ in sequence, contained polyps. These three calices were concentrated on the proximal side, while the edge of the colony contained only tubular and mesh‐like canals (Figure 2a). This suggests that mineralization for new skeleton growth and canal system formation precedes polyp budding and movement during coral growth. Vertical section S_1_ through S_3_ of the polyp‐canal system reconstruction depicts two large cavities within the tubular canals between calices P_1_ to P_2_ and P_2_ to P_3_. These transit areas are larger voids within the tubular canals, providing pathways for the newly budded polyps. Our observations suggest that these areas are key markers for polyp budding and play a crucial role in the directional growth and migration of polyps within the colony. While the exact mechanism of polyp movement within the colony remains unclear, our data suggest that newly budded polyps passively migrate through the canal system after their formation. The transit areas facilitate this migration, guiding polyps to their new calices. The polyp movement appears to be driven by the outward extension of the coenosarc tissue, which connects the polyps across the colony. These reconstructions indicate that these canals serve as transit areas for newly budded polyps moving between adjacent calices with budding relations. A similar canal is located near the base of calice P_3_ close to the colony edge, where a new polyp is presumed to emerge after budding.

Based on the reconstructions (Figures 1b and 2a), we can visualize the growth and budding process of the *M. capricornis* colony in the edge region. After the formation of skeletons with canal systems in the outermost region, new calices start to form while the base of the budding polyp begins constructing a large canal for transportation (Figure 2b). When the transit area connects the bases of these calices, the newly budded polyp can enter its new calice through the canal.

### 2.3 Transit Areas as Morphological Budding Markers in Coral Growth Analysis

Larger transit areas with distinct volume and structure exist between adjacent polyps with budding relationships, differing from the tubular canals (Figure 2). To better characterize the differences between transit areas and tubular canals, we performed voxel volume measurements using VG Studio Max 3.3. Transit areas were found to have a volume ranging from 1 mm^3^, with a skeleton‐to‐void ratio of 20%–35%. In contrast, tubular canals had smaller individual volumes of approximately 0.2 mm^3^ and a higher skeleton‐to‐void ratio of 45%–55%. This distinction in both volume and skeleton‐to‐void ratio confirms that transit areas are structurally unique compared to standard tubular canals. Such differences are crucial because they suggest that transit areas are specifically adapted to facilitate the movement of newly budded polyps.

**FIGURE 3:**
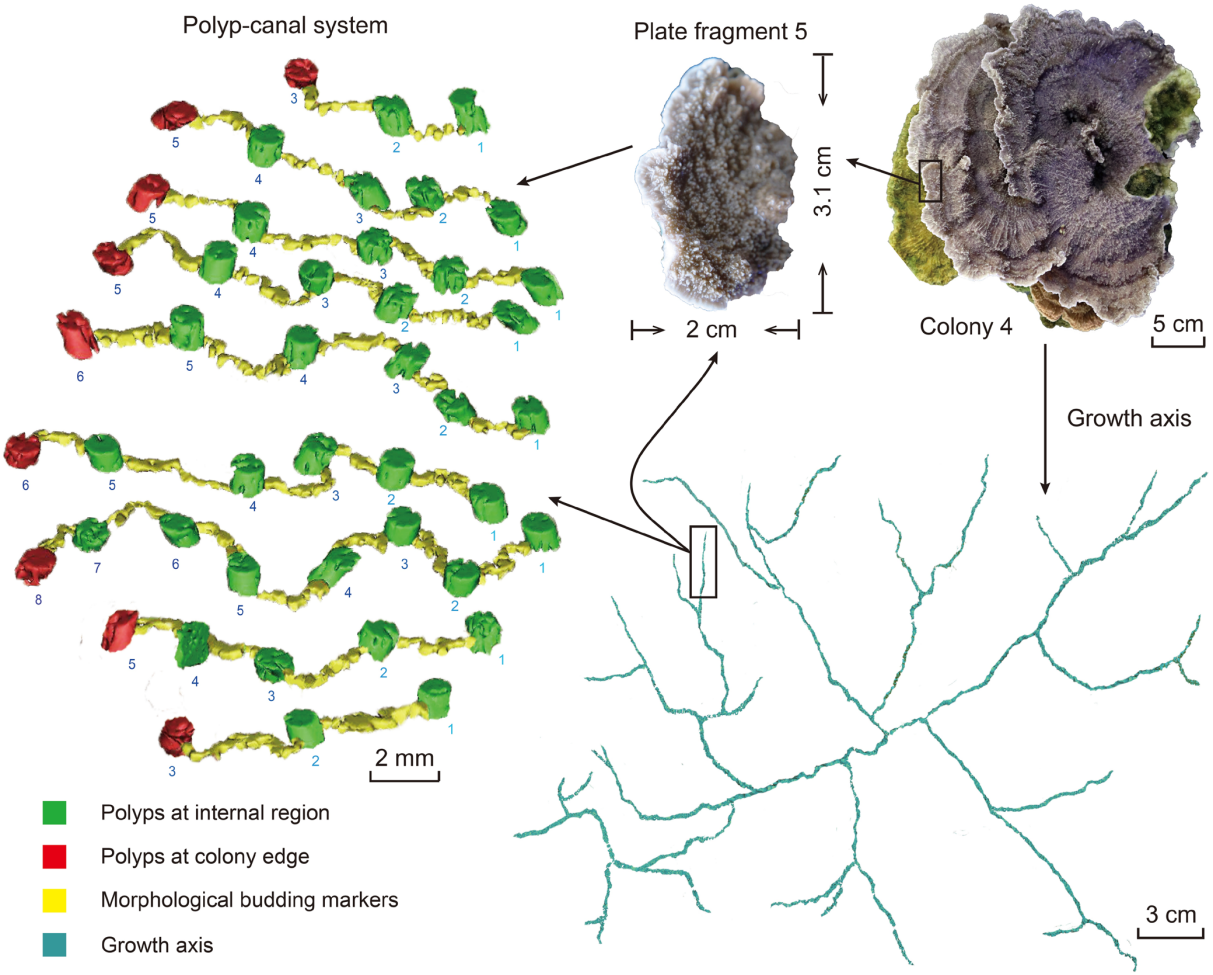
Reconstructions of polyp‐canal system and growth axis based on morphological budding markers. Owing to the structural differences, transit areas can be considered as morphological budding markers, utilized for highlighting adjacent polyps in the polyp‐canal system that exhibit budding relationships, thereby revealing the growth direction, budding trajectory, and distribution of polyps, and ultimately visualizing the growth axis of the entire coral colony. Budding trajectories are color‐coded according to their distance from the proximal origin of the fragment, from light blue to dark blue, to visualize the dynamic extension of the growth axis. Within a fan‐shaped coral fragment measuring 2 cm in width and 3.1 cm in length, a total of 46 polyps were observed. This method enables us to investigate the seasonal impact of external conditions on coral growth patterns.

These transit areas serve as morphological budding markers for investigating the growth patterns of *M. capricornis* . They are used to investigate coral growth patterns, including colony expansion direction, growth axis, polyp budding trajectory, and skeleton formation speed (Figure 3, Methods).

We reconstructed all polyps and budding markers within the plate fragments of the *M. capricornis* colony. Within the polyp‐canal system, the transit areas serving as budding markers formed a fan‐shaped network of budding trajectories connecting related polyps (Figure 3). These trajectories exhibited directional extension along zigzag lines, with varying spacing between adjacent budding trajectories, varied as the extension process unfolded, indicating a diverse pace of skeleton formation. The transit areas also displayed varying lengths, resulting in an uneven distribution of polyps.

The budding trajectories exhibit varying lengths, with those in closer proximity to the periphery of the fan‐shaped fragment being shorter and containing fewer polyps. The sixth and seventh budding trajectories from top to bottom harbor more polyps, positioning their outermost polyps closer to the growth point of the fragment. Consequently, these two budding trajectories tend to approach the growth axis of this fragment. This methodology also allows us to visualize the growth axis of the entire coral colony (Figure 3).

## 3 Discussion

### 3.1 Coral Skeleton Precedes the Polyps During Growth Process

Our 3D reconstructions of the skeleton–canal network in *M. capricornis* not only describe a new morphological budding marker but also reveal a specific growth strategy in which skeleton formation, canal remodeling, and polyp budding are tightly coupled in space and time.

Reconstructions show that *M. capricornis* growth begins with peripheral skeleton‐canal formation, followed by coenosarc extension and polyp budding (Figure 1b). On the side of the calice closest to the outer margin, a relatively large transit area was observed, which may facilitate the establishment of newly budded polyps in new calices, inferred from their spatial arrangement with the canal network (Figure 2a). This large‐volume transit area can be considered a morphological budding marker of *M. capricornis* . Simultaneously, in the area where the coenosarc tissue extends, new calices are constructed. Once the new calices are completed, the transit area connects the budding sites and the new calices, enabling the polyps to enter their new calices (Figure 2b).

Following the inferred establishment of new polyps, the transit area may integrate into the tubular canal, suggesting a structural assimilation, which warrants additional study. In this growth process, the initial stage involves the formation of a skeletal framework with intricate canal structures at the periphery, succeeded by the outward extension of coenosarc tissue through the internal canal network. This cyclic growth process likely involves the construction of the skeleton–canal network by coenosarc tissue, followed by polyp budding, with the transit areas potentially facilitating polyp establishment along specific canals, inferred from the observed structures (Li, Liao, et al. 2021). The establishment of large‐volume transit areas among calices represents an essential mechanism for facilitating budding and colonization by new polyps. This spatial arrangement ensures efficient transportation of newly budding polyps within coral colonies. Our findings on the skeleton–canal network and transit areas provide a structural framework that can be correlated with future molecular and genetic studies to understand the regulation of biomineralization and budding processes in corals, potentially informing protein expression and signal pathway analyses. These insights shed light on the complex biological processes underlying coral growth.

It is important to note that micro‐CT imaging primarily resolves the mineral skeleton and the internal canal space, whereas the soft tissues themselves remain below the resolution of our scans. Consequently, the position and deformation of the coenosarc and associated organs during polyp budding are inferred from the observed skeletal and canal structures rather than imaged directly. Future work that combines micro‐CT with complementary techniques, such as serial histology, in vivo fluorescence imaging, or contrast‐enhanced 3D tomography, will be essential to generate explicit anatomical reconstructions of the coenosarc during budding. Such integrated approaches would allow the type of detailed anatomical illustrations envisaged here and provide a more complete picture of the coupling between tissue dynamics and skeleton formation.

### 3.2 Skeleton Formation and Dissolution Coexist During Coral Growth

Visualization of the coral growth process reveals that during the formation of the skeleton‐canal system, not only does the emergence of new skeletons take place but also alterations in the skeletal structure and the dissolution of the original skeleton occur as a result of requirements such as budding, polyp translocation, calices construction, and the like. For instance, when transit areas for polyp budding and movement begin to emerge in tubular canals, the formation of calices and tubular canals commences at the outermost edge of the colony, where initially comprises mesh‐like canals within the skeleton only, and as the volume of new calices gradually increases to match that of other existing calices (Figures 1 and 2), there are processes involving diminution or structural alteration in the pre‐existing skeletal framework, potentially exerting simultaneous effects on extracellular calcifying medium (ECM). Matrix metalloproteinase 24, which belongs to the matrix metalloproteinase gene family, is hypothesized by researchers to potentially play a role in coral calcification and skeletal formation, and has the function of balancing and regulating the degradation of ECM proteins, or is related to this process (Xie et al. 2023). This indicates that the utilization of micro‐CT technology for reconstructing the skeletal–canal network can offer dynamic visual evidence to investigate coral growth and skeleton formation, and potentially facilitate research on coral biomineralization at the molecular level.

### 3.3 Identification and Implications of Budding Markers in *M. capricornis*

We identified morphological budding markers in the polyp‐canal system of *M. capricornis* (Figure 3). Specifically, during growth, the side of the calices where the budding polyp is located develops a larger canal region. This region gradually extends to the newly formed calices and connects to them, facilitating the transport of newly budded polyps (Figure 2a). After transport, this area integrates into the tubular canals with a distinct structure characterized by larger size and a smaller skeleton‐to‐void volume ratio (Figure 1b). Based on these budding markers, we can determine the budding relationships between coral polyps within *M. capricornis* colonies through micro‐CT and 3D reconstructions, allowing us to visualize the growth axis, trajectory of polyp movement, formation process of the skeleton–canal network, etc. (Figure 3). In contrast to the bamboo‐like calices of *Pocillopora* and *Stylophora*, where polyp movement trajectories are easily discernible from skeleton and canal structures (Li, Liao, et al. 2021), the intricate canal network of *M. capricornis* poses greater challenges for visualization. In contrast to the method, we proposed in our 2021 study (Li et al. 2023), which involved reconstructing canals in each branch of the colony from the proximal bifurcating point to the distal edge, canal reconstruction based on budding markers provides a more precise visualization of the growth axis of *M. capricornis* . Thus, tracing the polyp growth and budding trajectories in *M. capricornis* represents an advancement in understanding coral growth dynamics and skeletal formation processes, highlighting the breakthrough value of research on this complex genus.

Ecologically, markers quantify polyp distribution for transplantation metrics and model environmental impacts (e.g., temperature on growth axes) (Merks et al. 2004; Llabrés et al. 2024), informing seasonal resilience patterns and enhancing modular growth simulations under climate change. Our reconstruction results based on morphological budding markers serve as visual evidence for investigating the impacts of external factors on coral growth. This, combined with information on water flow, temperature variations, and light exposure at different stages, would allow us to analyze differences in the speed and pattern of polyp‐canal system construction under varying external environmental factors. Thus, we may gain insights into annual, seasonal, and monthly mechanisms influencing coral growth under varying external conditions. This method can also be employed to rapidly estimate the distribution of polyps per unit volume within coral colonies, providing quantitative metrics for coral transplantation (Figure 3). In conclusion, the reconstruction of budding markers and the polyp‐canal system along the growth axis enables us to reveal the cyclical growth patterns of reef‐building corals. The identification of transit areas as budding markers and the reconstruction of polyp‐canal trajectories provide a structural framework for understanding how colony‐scale growth directionality emerges from local interactions between skeleton formation, canal remodeling, and polyp budding. These insights are directly relevant to models of reef expansion and space occupation in reef‐building corals. In addition, the identified budding markers, particularly the transit areas, can be incorporated into existing coral growth models, such as those based on polyp replication (Merks et al. 2004; Llabrés et al. 2024). For instance, the spatial distribution and frequency of transit areas can be used to parameterize models of colony expansion, providing insights into modular growth dynamics under varying environmental conditions.

## 4 Methods

### 4.1 Sample Collection

The *M. capricornis* samples were collected from the Xisha Islands (15°40′–17°10′ N, 111°‐113° E) in 2018. Samples were collected from tropical shallow reefs at depths ranging from 5 to 10 m, with daily mean temperatures of 23.2°C to 29.2°C. The seawater pH at the collection site is 8.1–8.2, and the salinity is 34 ppt. Four whole colonies and five plate fragments were collected from different individuals to ensure genetic diversity. The fragments were collected from distinct colonies to avoid redundancy, and their morphological similarity was confirmed visually. The sampling interval is approximately 20 m. The approximate dimensions of each experimental sample are as follows: four *M. capricornis* colonies, 25 × 25 × 20 cm, 20 × 20 × 15 cm, 23 × 22 × 17 cm, and 25 × 20 × 15 cm; five *M. capricornis* fragments were collected from different coral colonies, but their morphological volume is similar, measuring 3 × 3 × 0.7 cm. Before the experiments, samples were maintained in a controlled laboratory environment. The tank was set to mimic natural conditions with a temperature of 26°C ± 1°C, salinity of 34 ppt, pH of 8.1–8.2, and a light cycle of 12 h light and 12 h dark with LED lights providing 200 photons m^2^/s. Scleractinian corals do not require feeding during the cultivation process.

### 4.2 Choice of Species and Identification

We selected *Montipora capricornis* for this study due to its ecological importance as a reef‐builder in the Indo‐Pacific region and the lack of detailed studies on its growth and budding mechanisms. The species was identified based on morphological characteristics as described by WoRMS and confirmed through genetic analysis based on the *M. capricornis* genome data (GCF_036669925.1) we previously uploaded to the National Center for Biotechnology Information (NCBI). Identification was based on key features such as colony growth form, polyp size, and skeletal structure, verified using traditional taxonomic methods and modern genetic techniques.

### 4.3 Micro‐CT Test

Coral samples were analyzed using 3D models constructed with a 230 kV latest‐generation X‐ray microfocus computed tomography system (Phoenix v|tome|x m; General Electric, GE; at Yinghua NDT, Shanghai, China). In our experiments, before scanning, colonies and fragments were briefly removed from the aquarium, excess water was blotted away, and samples were mounted in air on the micro‐CT stage without any additional fixation, dehydration, or embedding. The scanning parameters are detailed in Table 1, and reducing sample volume improves resolution. Two‐dimensional image reconstructions of each specimen from the matrices of scan slices were assembled using proprietary software from GE. The 3D reconstructions were performed using VG Studio Max 3.3, and the skeleton–canal network was visualized and analyzed to infer growth patterns based on structural variations observed in different parts of the colony. Given the single scan per sample, dynamic growth processes were inferred by comparing structural variations across multiple samples at different growth stages, as detailed in the subsequent section on dynamic process simulation. The micro‐CT experiment requires no sample preparation, enabling direct examination of living specimens while ensuring that all samples remain viable throughout the imaging process. This non‐destructive approach offers a distinct advantage for studying coral growth processes and patterns, preserving the natural state of the specimens during analysis.

### 4.4 3D Reconstructions of Skeleton, Polyp, and Canal

Slice data derived from the scans were then analyzed and manipulated using 3D reconstruction software. Reconstructions of the skeleton–canal network and polyp‐canal system were performed using VG Studio Max (v3.3.0). The 3D reconstructions were created following the method previously described (Li et al. 2020). Images of the reconstructions were exported from Mimics and VG Studio Max and finalized in Adobe Photoshop CC 2019 and Adobe Illustrator CC 2019. Vertical sections S_1_‐S_3_ in Figures were obtained as virtual slices in VG Studio Max by defining planes perpendicular to the colony surface and parallel to the local growth axis. These planes were positioned to intersect the centres of the calices of interest and the corresponding transit areas.

### 4.5 Simulation of Dynamic Coral Growth Processes

Static reconstructions (40–60 polyps in fragment, thousands in colony) were sequenced by structural progression (e.g., transit area emergence) to simulate temporality, validated in prior studies (Li et al. 2023) Repeatability was confirmed across five fragments, with potential for future time‐series confirmation (6–12 months).

### 4.6 Skeleton to Void Ratio Measurement

The skeletal matter‐to‐void volumetric ratio of coral samples, referred to as the “skeleton‐to‐void ratio,” was calculated in VG Studio Max 3.3 using surface determination, manual region selection, and porosity analysis. Transit areas (~1 mm^3^, 20%–35% ratio ±5% SD) differed from tubular canals (~0.2 mm^3^, 45%–55% ± 7% SD; *n* = 5). Detailed data are shown in Table 2. Error propagation accounted for voxel variability.

**TABLE 2:** Parameters of the transit areas and tubular canals.

Fragment	Type	ID	Volume (mm^3^)	Skeleton‐to‐void ratio (%)
Fragment 1	Transit area	1	1.05	26.33
		2	0.99	35.40
		3	1.06	31.34
		4	1.15	25.15
		5	0.98	30.21
	Tubular canal	1	0.19	46.06
		2	0.19	42.91
		3	0.20	52.20
		4	0.16	43.64
		5	0.17	40.11
Fragment 2	Transit area	1	1.15	28.05
		2	0.98	21.75
		3	1.01	29.38
		4	0.86	24.50
		5	0.95	26.04
	Tubular canal	1	0.19	41.45
		2	0.24	51.46
		3	0.20	36.28
		4	0.18	40.70
		5	0.22	51.38
Fragment 3	transit area	1	1.02	25.20
		2	1.07	23.90
		3	0.99	32.79
		4	0.97	29.22
		5	0.85	18.68
	Tubular canal	1	0.21	56.52
		2	0.19	44.13
		3	0.19	47.84
		4	0.21	52.32
		5	0.22	56.83
Fragment 4	transit area	1	0.95	34.28
		2	0.98	27.14
		3	0.89	32.52
		4	0.88	29.31
		5	1.08	24.27
	Tubular canal	1	0.21	55.75
		2	0.23	50.61
		3	0.20	47.91
		4	0.23	50.64
		5	0.15	36.09
Fragment 5	Transit area	1	0.98	24.99
		2	1.04	32.08
		3	1.15	29.14
		4	0.95	24.85
		5	0.92	30.07
	Tubular canal	1	0.20	39.76
		2	0.22	52.07
		3	0.19	51.83
		4	0.19	50.04
		5	0.19	48.36

## Author Contributions

**Yixin Li:** conceptualization (lead), data curation (lead), formal analysis (lead), funding acquisition (lead), investigation (lead), methodology (lead), project administration (lead), resources (lead), validation (lead), visualization (lead), writing – original draft (lead), writing – review and editing (lead). **Chenyi Wang:** supervision (lead), validation (supporting), writing – review and editing (supporting). **Zuhong Lu:** resources (supporting), software (equal), supervision (supporting), writing – review and editing (supporting). **Chuanliang Wu:** funding acquisition (supporting), writing – review and editing (supporting). **Chunpeng He:** resources (supporting), software (equal), supervision (supporting), writing – review and editing (supporting).

## Funding

This research is supported by Key Laboratory of Environment Controlled Aquaculture (Dalian Ocean University), Ministry of Education, China, 116023, grant number: KLECA202401; Joint Fund of the Natural Science Foundation of Liaoning Province (Doctoral Research Startup Project), grant number: 2023‐BSBA‐090; the Fundamental Research Funds for the Central Universities, grant number: DUT24BS070; and the Special Funding of Southeast University, grant number: 6907038067, 6907038068, and 6907038148.

## Ethics Statement

This work did not require ethical approval from a human subject or animal welfare committee.

## Conflicts of Interest

The authors declare no conflicts of interest.

## Data Availability

The HRCT data supporting this study's findings are available in Figshare through https://doi.org/10.6084/m9.figshare.20210720.v1.
